# Longitudinal associations between response-style strategies and abnormal eating behaviors/attitudes in adolescents: a cross-lagged panel model

**DOI:** 10.1186/s40337-024-00991-4

**Published:** 2024-02-27

**Authors:** Yasuo Murayama, Hiroyuki Ito, Megumi Hamada, Nobuya Takayanagi, Takahiro Nakajima, Mitsunori Myogan, Masatsugu Tsujii

**Affiliations:** 1 Institute of Human and Social Sciences, Kanazawa University, Kakuma-Machi, Kanazawa, Ishikawa 920-1192 Japan; 2 Faculty of Core Research Human Science Division, Ochanomizu University, 2-1-1, Otsuka, Bunkyo-ku, Tokyo, 112-8610 Japan; 3 School of Psychology, Chukyo University, 101-2, Yagoto Honmachi, Showa-ku, Nagoya, Aichi 466-8666 Japan; 4 Department of Psychology, Aichi University of Education, 1 Hirosawa, Igaya-Cho, Kariya, Aichi 448-8542 Japan; 5 School of Human Care Studies, Nagoya University of Arts and Sciences, 57, Takenoyama, Iwasaki-Cho, Nisshin, Aich 470-0196 Japan; 6 School of Contemporary Sociology, Chukyo University, 101, Tokodachi, Kaizu-Cho, Toyota, Aichi 470-0393 Japan; 7 Research Center for Child Mental Development, Hamamatsu University School of Medicine, 1-20-1, Hadayama, Higashi-Ku, Hamamatsu, Shizuoka 431-3192 Japan

**Keywords:** Response style, Disordered eating behavior/attitude, Rumination, Distraction, Depression, Cross-lagged panel model

## Abstract

**Background:**

Previous studies have suggested that response-style strategies (rumination, problem-solving, and distraction) can be risk or protective factors for the development of abnormal eating behaviors/attitudes (AEB) during adolescence. However, due to limited empirical data regarding the prospective effects of these strategies on AEB, further research is needed to clarify their role in developing AEB in adolescence.

**Methods:**

This study investigated the one-year lagged effects of response-style strategies on AEB in 24,883 fourth- to eighth-grade students in Japan between 2015 and 2019 using a cross-lagged panel model. Depressive symptoms and body mass index (BMI), which are reported to relate to AEB, were also included in the analytic model. The students self-reported their levels of response-style strategies, AEB, and depressive symptoms. We also evaluated BMI based on teachers’ reports.

**Results:**

We found that greater rumination significantly predicted more severe AEB in the following year among students from all grades, with small to moderate effect sizes. In addition, distraction significantly predicted more severe binge eating/purging behaviors, but with very weak small effect sizes. Problem-solving did not predict any level of AEB. Furthermore, we observed significant reciprocal relationships between response-style strategies, AEB, and depressive symptoms. Positive reciprocal associations between BMI and AEB were also found except for some intervals.

**Conclusions:**

We concluded that a decrease in rumination is critical to alleviating mental health problems, such as AEB and depressive symptoms, during adolescence. This suggests that interventions to reduce the level of rumination should be conducted in the early stages of adolescence.

*Trial Registration Number*: Not Applicable.

## Abstract (plain language summary)

This study examines the effects of three response styles when faced with distress—rumination, problem-solving, and distraction—on unhealthy eating behaviors. In this study, we looked at how these coping strategies are linked to unhealthy eating behaviors in 24,883 students in grades four to eight in Japan between 2015 and 2019. We checked again at year-long intervals to see if the coping strategies had any effect on the students’ unhealthy eating behaviors. In addition, we considered depressive symptoms and body mass index in our analysis because these variables might also be related to unhealthy eating behaviors. We found that more rumination (i.e., a repetitive negative thinking pattern) predicted the severity of unhealthy eating behaviors and depressive symptoms in the following year across all the grades. In addition, greater depressive symptoms also contributed to an increase in unhealthy eating behaviors in the following year. Therefore, we suggest that rumination is a key factor that influences mental health during adolescence.

## Background

Symptoms of psychopathology are likely to develop in adolescence [1]. One such symptomatology is abnormal eating behaviors/attitudes (AEB), which are symptoms of eating disorders [2]. Various studies have reported the prevalence of AEB among adolescents. For example, a study addressing a nationally representative adolescent sample in Australia showed that over 30% of adolescents experience AEB [3]. In the United States, more than 50% of adolescent girls and over 30% of adolescent boys reported engaging in at least one AEB [4]. Similarly, studies conducted in Eastern cultures have observed the prevalence of AEB among adolescents. In China, for instance, 35% of adolescent girls (*n* = 2019) and 14% of adolescent boys (*n* = 1525) surpassed the cutoff score on the bulimic subscale of the EDI-3 [5]. In Japan, approximately 20% of early adolescents were found to exhibit a high drive for thinness, a core symptom of anorexia [6]. Overall, these findings suggest that AEB is likely to develop during adolescence across different countries. Given the empirical evidence indicating that individuals with AEB are prone to experiencing mental health issues, functional impairment, and even mortality [2, 3], it is imperative to comprehend the factors that contribute to or deter the emergence of AEB during this crucial life stage.

Response-style strategies, which have been reported to be associated with the development of psychiatric symptoms during adolescence [7, 8], can function as either risk or protective factors for the development of AEB. While several theoretical frameworks, such as antecedent- and response-focused emotion regulation [9], have been proposed to understand emotion regulation, response style is one of the major frameworks. The response style is defined as a reaction to distress that contribute to its duration [10]. Nolen-Hoeksema [11] originally proposed that these styles include rumination, problem-solving, and distraction. These three strategies are widely recognized as major emotion regulation strategies [12, 13].

Rumination refers to the repetitive internal process of dwelling on distress and its possible causes and consequences without engaging in active problem-solving behaviors [14]. Several prospective studies have demonstrated that rumination exacerbates AEB in adolescents. For instance, a recent study found that rumination at a one-year lag predicted the severity of drive for thinness and bulimic symptoms among adolescents [15]. Similarly, other prospective studies have shown that greater rumination is associated with the severity of AEB [16, 17]. However, these studies did not control for the other two strategies, which are moderately associated with rumination levels [13, 18]; therefore, because the association between rumination and AEB may be mediated by the other strategies and may not accurately reflect the strength of the actual association, the reported associations may be spurious. Additional research is necessary to confirm these findings.

Problem-solving is a deliberate attempt to positively change a stressful situation or eliminate the adverse effects associated with it [12]. Classically, this strategy is referred to as “proper” problem-solving [19]. Cognitive-behavioral therapy targeting eating disorders (i.e., enhanced cognitive behavior therapy: CBT-E) incorporates some aspects of problem-solving strategies [20]. However, there is limited empirical data on the associations between this strategy and AEB. A meta-analysis of cross-sectional studies reported a weak negative association between problem-solving and AEB [12]. A recent cross-sectional study among adolescents reported a negative association of the frequency of using problem-solving with anorexic and bulimic symptoms [13]. However, regarding other psychopathological symptoms, more frequent use of problem-solving has been predicted to decrease depressive symptoms [21, 22]. Moreover, adolescents who use the problem-solving strategy more frequently show lower aggression [23].

Distraction involves engaging in pleasant activities to divert attention from distress [18]. Similar to problem-solving, we have limited empirical data on the associations between distraction and AEB. For example, more frequent use of distraction has been reported to be related to greater levels of anorexic and bulimic symptoms [13]. Another study demonstrated that more frequent use of distraction predicted greater bulimic, but not anorexic, symptoms among individuals diagnosed with eating disorders [24]. Regarding other psychopathological symptoms, cross-sectional studies have demonstrated a negative association between distraction and depressive symptoms [23, 25]. Prospective studies have shown controversial results, with one study reporting a positive effect of distraction on depressive symptoms [22], while another study failed to find such an effect [21]. To the best of our knowledge, few studies have examined the prospective associations of problem-solving and distraction with AEB during adolescence. Consequently, there is a lack of empirical data on the prospective associations between these three response-style strategies and AEB during adolescence.

To establish clear prospective associations between response-style strategies and AEB, it is imperative to consider potential confounding variables related to the levels of AEB. Notably, variables such as body mass index (BMI) and depressive symptoms, both known to be linked to AEB, should be carefully taken into consideration. For instance, one theory explaining the development of bulimic symptoms posits that successive relationships between BMI, components of anorexic symptoms (i.e., body dissatisfaction and dietary restraint), depressive symptoms, and other factors lead to the emergence of bulimic symptoms [26]. In fact, each of these prospective relationships has been supported empirically. For example, higher BMI has been found to elevate the risk for later body dissatisfaction [27] and bulimic symptoms [28]. Conversely, the relationship in the opposite direction has also been supported; adolescents with AEB have exhibited elevated BMI [4]. Moreover, several longitudinal studies focusing on adolescents have highlighted a reciprocal and positive relationship between depressive symptoms and bulimic symptoms [29–31]. Furthermore, prospective effects of response-style strategies on the development of depressive symptoms have been found [10, 32]. Considering these findings, it becomes evident that BMI and depressive symptoms may function as confounding variables, potentially obscuring the prospective associations between response-style strategies and AEB during adolescence.

In summary, little empirical data are available on the effects of response-style strategies on the severity of AEB in adolescents. Therefore, we aimed to simultaneously examine the effects of the three abovementioned response-style strategies on AEB among adolescents using the cross-lagged model, which included the response-style strategies, AEB, BMI, depressive symptoms, and demographic variables, such as socioeconomic status (SES). Prospective studies [15] and theories of psychological treatment for eating disorders (e.g., [20]) have demonstrated the prospective association between anorexic and bulimic symptoms. Therefore, using a single variable integrating anorexic and bulimic symptoms in the analytical model, we may fail to estimate accurate effect sizes regarding the response-style strategies on AEB. Consequently, we analyzed anorexic and bulimic symptoms separately in this study.

Building upon previous empirical findings that explore longitudinal and cross-sectional associations between response-style strategies and AEB, we posit that increased frequency of rumination and distraction will be predictive of more severe levels of AEB. Conversely, we hypothesize that a higher frequency of problem-solving will be associated with lower levels of AEB.

## Methods

### Participants

We obtained data from the Aichi School Cohort Study Project (ASCS-Project), which investigates the mechanisms through which children and adolescents internalize and externalize problems. Since 2007, the ASCS-Project has administered an annual survey to children and adolescents who attend all public nursery, elementary, and junior high schools in a city in Aichi Prefecture, Japan. This study used data from annual surveys conducted between 2015 and 2019 on students in the fourth to ninth grades, the only students required to answer the items regarding the response-style strategies in the ASCS-Project.

A total of 24,883 students (12,505 boys and 12,378 girls) from the fourth to ninth grades were involved in this study. We addressed students from eight different cohorts (i.e., students who enrolled in elementary school from 2008 to 2015) (Table 1). Participants took annual surveys two to five times because, within the five-year study period, some students enrolled in the fourth grade or moved to the city where the ASCS-Project was administered. Other students moved out of the city or graduated from junior high schools. In the Japanese school system, almost no elementary and junior high school students fail to move on to the next grade, suggesting that the grades indicated by the students in the survey likely corresponded to their age (i.e., the students in fourth to ninth grades were 10–15 years old).

**Table 1:** Sample composition

Cohort Number	Years in which the surveys were conducted															
	2015			2016			2017			2018			2019			Total
	Grade	n		Grade	n		Grade	n		Grade	n		Grade	n		
		Boys	Girls		Boys	Girls		Boys	Girls		Boys	Girls		Boys	Girls	
1	8	447	450	9	446	452										1795
2	7	492	418	8	492	415	9	492	410							2719
3	6	414	467	7	394	454	8	399	441	9	396	441				3406
4	5	463	423	6	466	428	7	452	432	8	434	411	9	439	416	4364
5	4	443	446	5	446	453	6	448	454	7	414	427	8	408	421	4360
6				4	469	472	5	463	471	6	457	468	7	435	454	3689
7							4	462	465	5	460	466	6	456	463	2772
8										4	457	426	5	461	434	1778
Total		2259	2204		2713	2674		2716	2673		2618	2639		2199	2188	24,883

Cohort numbers represent different years in which the participants enrolled in school. For example, the participants in Cohort #1 enrolled in school in 2008 (i.e., these students were in eighth grade in 2015 and ninth grade in 2016). Similarly, the participants in Cohorts #2 to #8 enrolled in school from 2009 to 2015, respectively

### Measures

#### Response-style strategies

We used the Response Styles Questionnaire for Middle School Students (RSQ-MS) [33] to measure how frequently the students used response-style strategies in their daily lives. The RSQ-MS was developed from the items of the original RSQ [34], the Children’s RSQ [18], and the Japanese version of the RSQ [35]. The RSQ-MS is a 16-item questionnaire grouped into four subscales: rumination (four items), problem-solving (five items), distraction (three items), and escape from thinking (four items). Given that the response styles theory and the original RSQ do not include the strategy to escape from thinking, and the subscale for this strategy has been reported to show low validity [33], this study used only the first three subscales adapted from the original RSQ. Individuals rated their answers using a four-point Likert scale (1 = *almost never*, 4 = *almost always*), with higher scores indicating more frequent use of the strategies. The RSQ-MS except the subscale of escape from thinking has been reported to have good reliability and validity among Japanese adolescents [33]. In the current study, MacDonald’s Omega was found to be 0.790–0.836, 0.827–0.840, and 0.724–0.728 for the rumination, problem-solving, and distraction subscales, respectively.

#### Abnormal eating behaviors/attitudes

We used the Abnormal Eating Behavior Questionnaire for Elementary and Junior High School Students (ABQ-EJ) [36] to assess the severity of students’ AEB. This self-reported scale contains 14 items grouped into two subscales. The drive for thinness subscale comprises eight items related to body dissatisfaction and excessive dietary restriction, which are key symptoms of anorexia nervosa. The bulimic symptoms subscale includes five items regarding binge eating and purging behaviors. Adolescents rated their answers using a four-point Likert scale (1 = *almost never*, 4 = *almost always*), with higher scores indicating more severe AEB. An empirical study targeting Japanese adolescents reported that the ABQ-EJ has good reliability and validity [36]. MacDonald’s Omega was 0.813–0.823 for the drive for thinness subscale and 0.748–0.779 for the bulimic symptom subscale in our sample.

#### Depressive symptoms

Empirical data have indicated that depressive symptoms are prospectively and cross-sectionally associated with emotional regulation strategies and AEB [17], suggesting that depressive symptoms may be a confounding variable in this study. Therefore, we controlled for depressive symptoms in the proposed analytic model (more details are given in Subsection 2.4—Data Analysis). We used the Japanese short version of the Birleson Depression Self-Rating Scale for Children (JS-DSRS-C) [37], developed from the original version of the DSRS-C [38], to measure the level of participants’ depressive symptoms. The JS-DSRS-C comprises nine items. Respondents rate their answers using a four-point Likert scale (1 = *never*, 4 = *very often*). Higher scores indicate more severe depressive symptoms. The JS-DSRS-C has been reported to have good reliability and validity among Japanese adolescents [37]. MacDonald’s Omega was 0.783 –0.811 in the current study.

#### Body mass index (BMI)

Empirical research has indicated a prospective association between BMI and AEB [28]. Therefore, this study considered BMI as a potential confounding variable (details are provided in Subsection 2.4—Data Analysis below). Students' BMI was calculated using their height and body weight, which were measured annually during the school's physical examination conducted from May to June. The class teachers reported the height and body weight of each student for the current study.

#### Socioeconomic status

We used the Non-Intrusive Measurement of Socioeconomic Status questionnaire (NIMSES) [39] to measure the SES of the students’ families. The participants’ parents answered the NIMSES questionnaire for each survey during the five-year research period. The NIMSES contains 14 items that indirectly measure SES, including lifestyle. One empirical study addressing a sample of Japanese parents of adolescents reported that the NIMSES has good reliability and validity [39]. Each item is rated on a Likert scale ranging from 0 to 100, with higher scores indicating greater SES. MacDonald’s Omega was 0.684–0.701 in the current study.

### Procedure

The students’ parents were required to complete a consent form each year to allow their children to participate in the ASCS-Project. Only students whose parents had approved their participation responded to the questionnaires in class. Additionally, the parents who agreed to participate in the ASCS-Project answered the items measuring SES at home. The annual investigation was conducted in September every year. The study protocol was approved by the ethics board of Chukyo University (approval number: E14-328–1).

This study assessed data at one-year intervals, guided by the following evidence. First, empirical data have shown that the frequency of using response-style strategies can vary within a year during adolescence [10]. Second, AEB levels and BMI are likely to increase during adolescence [1, 3]. In addition, a household’s SES level may change every year due to family issues, such as parents changing careers. Hence, a prospective study with a longer interval between surveys (such as three years) may fail to uncover the relationships between response-style strategies and AEB.

### Data analysis

First, we assessed Pearson product-moment correlations to examine the cross-sectional and one-year-lagged relationships between response-style strategies, AEB, and the other variables. We interpreted the effect sizes of the correlation coefficients using Cohen’s criterion [40]. To examine the prospective effects of the three response-style strategies on AEB, we employed a structural approach based on a cross-lagged panel model including the response-style strategies, AEB, BMI, depressive symptom, and demographic variables using Mplus version 7 [41]. We have employed the cross-lagged panel model to analyze the prospective effects of between-person inferences on the recommendation of prior studies [42]. We included the demographic variables (i.e., gender, grade levels, and the level of SES) as covariates in the analytical model.

In addition, we controlled for cohort effects. Some previous research has noted that controlling for cohort effects in prospective studies allows for more robust and less biased results because cohorts and the times of the surveys are often confounded [43]. Recent studies on adolescents have revealed that cohort effects exist at the level of body shape and depressive symptoms [43, 44].

**Fig. 1:**
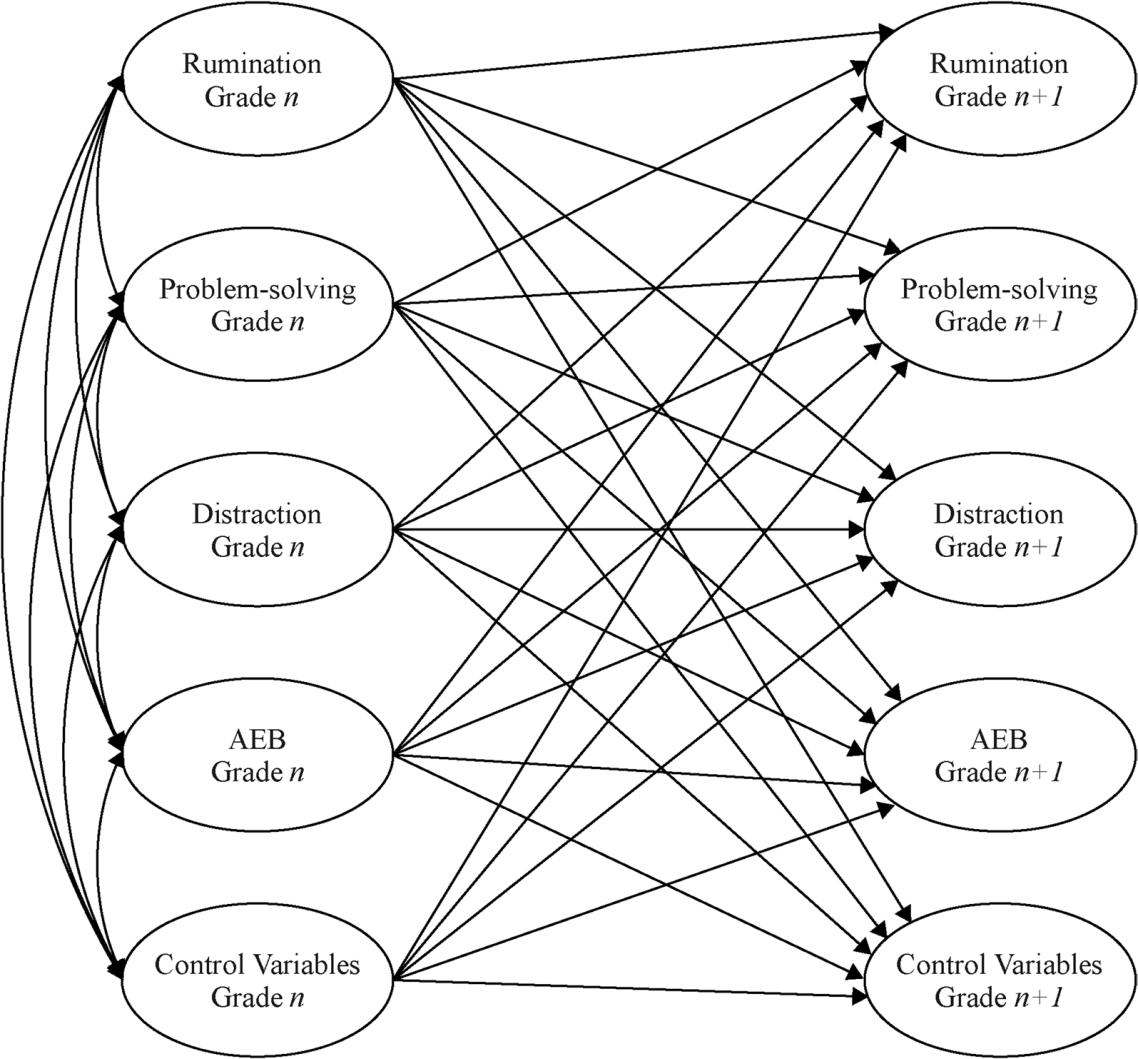
The cross-lagged model in the current study. *Note*. To simplify, contemporaneous correlations between each variable at Grade n and Grade *n* + 1 are not shown. *AEB* Abnormal eating behaviors/attitudes. Control variables include depressive symptoms, *BMI* Gender, grade, SES, and cohort (year of enrollment). A number between 4 and 8 will be assigned to *n*

Taking advantage of the ASCS-Project annual survey, we examined the one-year lagged effects of the response-style strategies on AEB among students from eight different cohorts. In other words, we estimated these effects between each pair of grades (i.e., between fourth and fifth grade, fifth and sixth grade, sixth and seventh grade, seventh and eighth grade, and eighth and ninth grade) by using data from the five surveys conducted from 2015 to 2019. Figure 1 illustrates the proposed analytical model.

Given that the coefficients of cross-lagged effects are smaller than those of multiple regression analysis, a guideline for interpreting the effect size of cross-lagged effects has recently been proposed (small: 0.03, medium: 0.07, large: 0.12) [43, 45]. Using this criterion, we interpreted the effect sizes of the one-year lagged effects. We standardized all quantitative data and used dummy or ordinal variables for qualitative data (i.e., gender and cohort). We handled missing data using full information maximum likelihood estimation, which is likely to provide less biased and more reliable results than alternative methods [46]. Finally, we used several goodness-of-fit measures, including RMSEA, CFI, TLI, and SRMR, to examine the fit of the model. We considered a model fit satisfactory if TLI and CFI were greater than or equal to 0.95, and RMSEA and SRMR were less than or equal to 0.06 and 0.07, respectively, according to Hu and Bentler’s criteria [47].

## Results

### Retention

The retention rates ranged between 96.7 and 98.6% in the five one-year intervals (i.e., between fourth and fifth grade, fifth and sixth grade, sixth and seventh grade, seventh and eighth grade, and eighth and ninth grade). We observed significant differences in some variables measured in each annual survey between the dropout students and retained students. Across all intervals, the dropout students exhibited greater depressive symptoms compared to the retained students (*t* = 4.43–5.91, *p*s < 0.001). Furthermore, in comparison to the retained students, the dropout students demonstrated higher levels of rumination (*t* = 2.24, *p* < 0.05), drive for thinness (*t* = 2.45–4.25, *p*s < 0.05), binge eating/purging behaviors (*t* = 2.10–2.71, *p*s < 0.05), and BMI (*t* = 2.22–2.68, *p*s < 0.05), as well as lower levels of problem-solving (*t* = 2.03–2.75, *p*s < 0.05) and distraction (*t* = 2.10–3.41, *p*s < 0.05) in certain intervals (Table 2).

**Table 2:** Significant differences in variables observed between dropout and retained students

Comparison	Variable	Intervals where significant differences were found
Dropout > Retained	Depressive symptoms	All intervals
	Rumination	G4—> G5
	BMI	G4—> G5, G5—> G6, G6—> G7, G7—> G8
	Drive for thinness	G6—> G7, G7—> G8
	Binge eating/purging	G5—> G6, G6—> G7, G7—> G8
Dropout < Retained	Problem-solving	G5—> G6, G7—> G8, G8—> G9
	Distraction	G7—> G8, G8—> G9

*G* Grade

### Correlations

Table 3 shows the correlations between the variables measured at Grade *n*. Rumination was positively correlated with drive for thinness and binge eating/purging behaviors, with small to medium effect sizes (drive for thinness: *r* = 0.259–0.313, binge eating/purging behaviors: *r* = 0.211–0.227). The coefficients of the correlations between problem-solving and drive for thinness and binge eating/purging behaviors were below 0.10, indicating small effects (drive for thinness: *r* = -0.024–0.009, binge eating/purging behaviors: *r* = 0.009–0.062). The correlation of distraction with drive for thinness and binge eating/purging behaviors was also small (drive for thinness: *r* = − 0.088–0.053, binge eating/purging behaviors: *r* = 0.053–0.193).

**Table 3:** Pearson’s product-moment correlations between variables measured at Grade *n*

		1	2	3	4	5	6
1	Problem-solving	–					
2	Rumination	.163 –.212	–				
3	Distraction	.395–.416	− .060 –.068	–			
4	Drive for thinness	− .024–.009	.259–.313	− .088–.053	–		
5	Binge eating/purging	.009–.062	.211–.227	.053–.193	.355 –.514	–	
6	Depressive symptoms	− .247–−.311	.394–.434	− .429–− .267	.215–.291	.099–.133	–
7	SES	.061–.106	− .018–.007	− .047–− .021	− .103–− .052	− .126–− .041	− .063–− .006
8	Sex (ref. = boy)	.043–.066	.028–.151	− .119– − .033	.007–.353	− .160– − .003	.035–.067
9	BMI	− .050–− .020	− .007–.045	− .030–.003	.236–.307	.125–.196	.009–.105
10	Cohort (Ref. = the lowest Cohort #)	.042–106	− .014–.039	− .073–0.09	− .324–.702	− .144–.371	− .034–.068

*SES* Socioeconomic status, BMI: body mass index. The absolute value of the coefficients above .0291, .0427, and 0545 is the 5%, 1%, and 0.1% significance levels (i.e., *p* < .05, .01, and .001), respectively

Table 4 shows the correlations between the variables measured at Grade *n* and those measured at Grade *n* + *1*. Rumination at Grade *n* was positively correlated with the levels of drive for thinness and binge eating/purging behaviors at Grade *n* + *1*, with small to medium effect sizes (drive for thinness: *r* = 0.166–0.272, binge eating/purging behaviors: *r* = 0.160–0.187). Regarding the correlations between problem-solving at Grade *n* and AEB at Grade *n* + *1*, the coefficients were below 0.10 (drive for thinness: *r* = − 0.043–0.034, binge eating/purging behaviors: *r* = − 0.006–0.040). Distraction at Grade *n* was also weakly correlated with the levels of AEB at Grade *n* + *1* (drive for thinness: *r* = − 0.069–0.035, binge eating/purging behaviors: *r* = 0.029–0.140).

**Table 4:** Pearson’s product-moment correlations between variables at Grade *n* and variables at Grade *n* + *1*

		Grade *n*						
		Problem solving	Rumination	Distraction	Drive for thinness	Binge eating/purging	Depressive Symptom	BMI
Grade *n* + *1*	Problem- solving	.419–.520	.037–.095	.201–.254	.005–.049	− .022 –.020	− .254–− .199	− .054–− .024
	Rumination	.065 –.104	.500–.657	− .088–− .006	.172–.287	.134–.186	.297– 373	− .002 –.045
	Distraction	.229–.249	− .125 – -.055	.397 –.561	− .069 – -.001	.022 –.080	− .318–− .258	− .023–.042
	Drive for thinness	− .043–.034	.166–.272	-.069–.035	.566–.692	.220–.388	.207–.262	.221–.281
	Binge eating/purging	− .006 –.040	.160–.187	.029–.140	.214–.357	.571–.597	.088–.118	.106–.170
	Depressive symptom	− .257 – − .170	.324–.358	− .164– − .318	.206–.251	.096–.110	.567–.705	.075–.108
	BMI	-.042 – − .012	.003 –.052	− .031–.019	.257–.303	.143–.213	.074–.094	.834–.872

The absolute value of the coefficients above .0291, .0427, and .0545 is the 5%, 1%, and 0.1% significance levels (i.e., *p* < .05, .01, and .001), respectively

### Cross-lagged effects of response-style strategies on AEB

The model fit the data well, even though the value of TLI was slightly below 0.95 (RMSEA = 0.030 [95% CI 0.029 –0.031], TLI = 0.934, CFI = 0.960, and SRMR = 0.045). Tables 5 and 6 display the results of the cross-lagged panel model addressing the effects of response-style strategies on drive for thinness and binge eating/purging behaviors, which are the main results of this study. Tables 7, 8 and 9 present the cross-lagged effects on the three response styles, while Table 10 and 11 indicate the effects on the control variables: depressive symptoms and BMI. Table 12 summarizes the main results related to the 1-year lagged effects, including the directions of the coefficients and the magnitudes of their effect sizes).

**Table 5:** Results of cross-lagged panel model regarding the effect of response-style strategies on the severity of drive for thinness

IVs (Grade n)	Intervals														
	G4 → G5			G5 → G6			G6 → G7			G7 → G8			G8 → G9		
	*β*	*SE*	*p*	*β*	*SE*	*p*	*β*	*SE*	*p*	*β*	*SE*	*p*	*β*	*SE*	*p*
Sex (ref. = boy)	.064	.011	.000	.097	.011	.000	.137	.011	.000	.192	.011	.000	.190	.012	.000
Cohort (ref. = the lowest Cohort #)	.135	.020	.000	.178	.018	.000	.129	.019	.000	.043	.021	.040	− .383	.023	.000
SES	− .018	.011	.111	− .058	.011	.000	− .047	.012	.000	− .005	.013	.678	− .048	.014	.001
BMI	.102	.011	.000	.119	.011	.000	.092	.011	.000	.086	.012	.000	.090	.012	.000
Depressive symptoms	.073	.010	.000	.063	.008	.000	.047	.006	.000	.029	.008	.001	.009	.012	.453
Problem-solving	.001	.010	.922	− .002	.007	.754	− .006	.006	.351	− .009	.008	.244	− .012	.010	.265
Rumination	.014	.010	.145	.029	.008	.000	.045	.006	.000	.060	.008	.000	.071	.011	.000
Distraction	− .011	.009	.256	− .003	.007	.712	.007	.006	.275	.016	.008	.040	.023	.010	.024
Drive for thinness	.522	.018	.000	.510	.012	.000	.534	.011	.000	.556	.011	.000	.568	.012	.000
Binge eating/purging	.049	.011	.000	.034	.008	.000	.021	.006	.000	.009	.007	.201	− .003	.010	.761

*SES* Socioeconomic status, *BMI* Body mass index

**Table 6:** Results of cross-lagged panel model regarding the effect of response-style strategies on the severity of binge eating/purging in Grade *n* + 1

IVs (Grade n)	Intervals														
	G4 → G5			G5 → G6			G6 → G7			G7 → G8			G8 → G9		
	*β*	*SE*	*p*	*β*	*SE*	*p*	*β*	*SE*	*p*	*β*	*SE*	*p*	*β*	*SE*	*p*
Sex (ref. = boy)	− .031	.012	.013	− .014	.012	.265	.013	.013	.314	.030	.013	.022	.013	.014	.357
Cohort (ref. = the lowest Cohort #)	.042	.024	.084	.044	.023	.053	.037	.022	.096	.023	.024	.324	− .160	.028	.000
SES	− .004	.013	.724	− .011	.013	.381	− .050	.013	.000	− .006	.014	.658	− .005	.016	.735
BMI	.060	.013	.000	.057	.013	.000	.019	.013	.143	.001	.013	.929	.040	.014	.004
Depressive symptoms	.039	.012	.001	.035	.009	.000	.028	.007	.000	.021	.009	.025	.014	.013	.295
Problem-solving	− .009	.011	.443	− .009	.008	.294	− .008	.007	.246	− .007	.009	.399	− .007	.012	.575
Rumination	.030	.012	.010	.033	.009	.000	.034	.007	.000	.035	.009	.000	.035	.012	.004
Distraction	.024	.011	.029	.026	.008	.002	.024	.007	.000	.023	.009	.007	.021	.011	.062
Drive for thinness	.024	.018	.180	.019	.011	.090	.015	.008	.078	.012	.010	.230	.010	.013	.473
Binge eating/purging	.534	.014	.000	.530	.010	.000	.537	.009	.000	.552	.009	.000	.596	.011	.000

*SES* Socioeconomic status, *BMI* Body mass index

**Table 7:** Results of cross-lagged panel model regarding the effect of response-style strategies on the severity of rumination in Grade n + 1

IVs (Grade n)	Intervals														
	G4 → G5			G5 → G6			G6 → G7			G7 → G8			G8 → G9		
	*β*	*SE*	*p*	*β*	*SE*	*p*	*β*	*SE*	*p*	*β*	*SE*	*p*	*β*	*SE*	*p*
Sex (ref. = boy)	.037	.012	.002	.051	.011	.000	.059	.012	.000	.053	.012	.000	.049	.013	.000
Cohort (ref. = the lowest Cohort #)	− .070	.020	.000	− .029	.017	.082	− .050	.017	.003	− .022	.020	.267	− .005	.027	.859
SES	.006	.012	.646	− .005	.012	.671	.015	.013	.246	− .002	.014	.901	− .014	.015	.351
BMI	− .028	.013	.030	− .015	.012	.191	.002	.012	.879	− .018	.012	.145	− .015	.013	.253
Depressive symptoms	.110	.011	.000	.105	.008	.000	.096	.007	.000	.091	.009	.000	.083	.013	.000
Problem-solving	.030	.012	.011	.030	.008	.000	.029	.007	.000	.028	.008	.000	.028	.011	.013
Rumination	.442	.011	.000	.485	.009	.000	.510	.008	.000	.554	.009	.000	.594	.011	.000
Distraction	− .023	.011	.043	− .021	.008	.008	− .018	.006	.004	− .016	.008	.042	− .014	.011	.207
Drive for thinness	.077	.019	.000	.063	.011	.000	.057	.008	.000	.054	.009	.000	.053	.013	.000
Binge eating/purging	.028	.013	.040	.023	.008	.007	.020	.006	.002	.019	.008	.012	.019	.011	.094

*SES* Socioeconomic status, *BMI* Body mass index

**Table 8:** Results of cross-lagged panel model regarding the effect of response-style strategies on the frequency of problem-solving in Grade *n* + 1

IVs (Grade n)	Intervals														
	G4 → G5			G5 → G6			G6 → G7			G7 → G8			G8 → G9		
	*β*	*SE*	*p*	*β*	*SE*	*p*	*β*	*SE*	*p*	*β*	*SE*	*p*	*β*	*SE*	*p*
Sex (ref. = boy)	.019	.013	.127	.044	.012	.000	.029	.012	.018	.027	.013	.044	.031	.015	.042
Cohort (ref. = the lowest Cohort #)	.055	.021	.010	.055	.018	.002	.070	.018	.000	.026	.022	.247	− .024	.031	.430
SES	.050	.013	.000	.058	.013	.000	.051	.014	.000	.039	.015	.010	.039	.017	.023
BMI	− .012	.014	.366	− .014	.013	.253	− .015	.013	.229	− .014	.014	.301	− .007	.015	.619
Depressive symptoms	− .105	.012	.000	− .107	.009	.000	− .109	.007	.000	− .112	.010	.000	− .116	.014	.000
Problem-solving	.390	.012	.000	.409	.010	.000	.433	.008	.000	.444	.010	.000	.475	.013	.000
Rumination	.020	.013	.108	.024	.009	.007	.028	.007	.000	.033	.009	.000	.039	.013	.004
Distraction	.026	.012	.030	.024	.009	.005	.021	.007	.002	.019	.009	.033	.016	.013	.195
Drive for thinness	.012	.020	.529	.011	.012	.338	.011	.009	.200	.012	.010	.251	.013	.015	.385
Binge eating/purging	− .039	.014	.007	− .025	.009	.006	− .015	.007	.028	− .007	.008	.415	.002	.013	.864

*SES* Socioeconomic status, *BMI* Body mass index

**Table 9:** Results of cross-lagged panel model regarding the effect of response-style strategies on the frequency of distraction in Grade *n* + 1

IVs (Grade n)	Intervals														
	G4 → G5			G5 → G6			G6 → G7			G7 → G8			G8 → G9		
	*β*	*SE*	*p*	*β*	*SE*	*p*	*β*	*SE*	*p*	*β*	*SE*	*p*	*β*	*SE*	*p*
Sex (ref. = boy)	− .077	.013	.000	− .039	.012	.001	− .011	.012	.375	.004	.013	.774	− .014	.015	.332
Cohort (ref. = the lowest Cohort #)	.003	.021	.871	− .025	.018	.153	− .040	.018	.021	− .012	.022	.570	− .115	.029	.000
SES	− .010	.013	.432	− .037	.013	.004	− .036	.014	.009	− .036	.015	.015	− .029	.017	.078
BMI	− .022	.013	.094	− .007	.013	.560	− .013	.013	.304	.019	.013	.141	− .013	.014	.384
Depressive symptoms	− .122	.012	.000	− .127	.009	.000	− .129	.007	.000	− .137	.009	.000	− .144	.014	.000
Problem-solving	.070	.012	.000	.056	.009	.000	.041	.007	.000	.026	.009	.002	.011	.013	.369
Rumination	− .051	.012	.000	− .046	.009	.000	− .040	.007	.000	− .035	.009	.000	− .029	.013	.027
Distraction	.350	.012	.000	.393	.009	.000	.417	.008	.000	.455	.009	.000	.492	.012	.000
Drive for thinness	.021	.020	.289	.017	.012	.147	.015	.009	.074	.015	.010	.134	.015	.014	.297
Binge eating/purging	.014	.014	.316	.016	.009	.082	.018	.007	.008	.022	.008	.007	.027	.012	.027

*SES* Socioeconomic status, *BMI* Body mass index

**Table 10:** Results of cross-lagged panel model regarding the effect of response-style strategies on the severity of depressive symptoms in Grade *n* + 1

IVs (Grade n)	Intervals														
	G4 → G5			G5 → G6			G6 → G7			G7 → G8			G8 → G9		
	*β*	*SE*	*p*	*β*	*SE*	*p*	*β*	*SE*	*p*	*β*	*SE*	*p*	*β*	*SE*	*p*
Sex (ref. = boy)	.026	.010	.009	.029	.010	.004	.006	.011	.608	.030	.011	.009	-.012	.013	.363
Cohort (ref. = the lowest Cohort #)	− .026	.015	.094	− .026	.013	.042	− .060	.015	.000	.025	.019	.182	.050	.025	.047
SES	− .023	.011	.031	− .016	.011	.162	.009	.012	.457	− .009	.013	.505	.018	.014	.217
BMI	.023	.011	.042	.013	.011	.214	.041	.011	.000	.004	.012	.735	.002	.013	.862
Depressive symptoms	.481	.010	.000	.522	.008	.000	.543	.007	.000	.588	.008	.000	.630	.011	.000
Problem-solving	− .072	.011	.000	− .071	.008	.000	− .067	.006	.000	− .065	.008	.000	− .064	.011	.000
Rumination	.127	.011	.000	.118	.008	.000	.106	.006	.000	.098	.008	.000	.090	.011	.000
Distraction	− .021	.011	.051	− .024	.008	.002	− .025	.006	.000	− .027	.008	.000	− .029	.010	.006
Drive for thinness	.090	.018	.000	.062	.010	.000	.044	.008	.000	.032	.009	.000	.022	.012	.067
Binge eating/purging	− .018	.013	.172	− .010	.008	.224	− .004	.006	.530	.001	.007	.859	.006	.010	.533

*SES* Socioeconomic status, BMI: body mass index

**Table 11:** Results of the cross-lagged panel model regarding the effects on BMI in Grade *n* + 1

IVs (Grade n)	Intervals														
	G4 → G5			G5 → G6			G6 → G7			G7 → G8			G8 → G9		
	*β*	*SE*	*p*	*β*	*SE*	*p*	*β*	*SE*	*p*	*β*	*SE*	*p*	*β*	*SE*	*p*
Sex (ref. = boy)	.000	.008	.965	.006	.007	.419	− .010	.007	.192	.017	.008	.031	.017	.009	.058
Cohort (ref. = the lowest Cohort #)	− .073	.013	.000	− .036	.010	.001	.042	.010	.000	− .008	.013	.540	.032	.018	.079
SES	− .005	.008	.530	.003	.008	.668	.007	.008	.394	− .007	.010	.460	− .023	.018	.033
BMI	.810	.005	.000	.873	.004	.000	.848	.004	.000	.851	.004	.000	.863	.005	.000
Depressive symptoms	.021	.007	.006	.014	.006	.012	.007	.005	.150	− .001	.006	.925	− .009	.009	.317
Problem-solving	− .001	.008	.870	.003	.006	.655	.006	.005	.186	.010	.006	.079	.014	.008	.080
Rumination	− .027	.008	.000	− .021	.006	.000	− .015	.005	.002	− .008	.006	.166	− .001	.008	.900
Distraction	.014	.008	.068	.011	.006	.039	.009	.005	.057	.006	.007	.263	.004	.008	.627
Drive for thinness	.097	.012	.000	.064	.007	.000	.044	.006	.000	.029	.007	.000	.014	.009	.113
Binge eating/purging	.029	.009	.001	.025	.006	.000	.023	.005	.000	.022	.005	.000	.023	.008	.003

*SES* Socioeconomic status, *BMI* Body mass index

**Table 12:** Summary on the main results of the cross-lagged model

direction of the relationship	Intervals						
		G4 → G5	G5 → G6	G6 → G7	G7 → G8	G8 → G9	
Rumination	→	Drive for thinness	–	P/S	P/S	P/S	P/M
	→	Binge eating/purging	P/S	P/S	P/S	P/S	P/S
	→	Depressive symptoms	P/L	P/L	P/M	P/M	P/M
Problem-solving	→	Drive for thinness	–	–	–	–	–
	→	Binge eating/purging	–	–	–	–	–
	→	Depressive symptoms	N/M	N/M	N/M	N/M	N/S
Distraction	→	Drive for thinness	–	–	–	P/U	P/U
	→	Binge eating/purging	P/U	P/U	P/U	P/U	–
	→	Depressive symptoms	–	N/U	N/S	N/S	N/S
Drive for thinness	→	Rumination	P/M	P/S	P/S	P/S	P/S
	→	Problem-solving	–	–	–	–	–
	→	Distraction	–	–	–	–	–
	→	Depressive symptoms	P/M	P/S	P/S	P/S	P/U
	→	BMI	P/M	P/S	P/S	P/S	–
Binge eating/purging	→	Rumination	P/S	P/U	P/U	P/U	–
	→	Problem-solving	N/S	N/U	N/U	–	–
	→	Distraction	–	–	P/U	P/U	P/U
	→	Depressive symptoms	–	–	–	–	–
	→	BMI	P/S	P/S	P/U	P/U	P/U
Depressive symptoms	→	Rumination	P/M	P/M	P/M	P/M	P/M
	→	Problem-solving	N/M	N/M	N/M	N/M	N/L
	→	Distraction	N/L	N/L	N/L	N/L	N/L
	→	Drive for thinness	P/M	P/S	P/S	P/S	–
	→	Binge eating/purging	P/S	P/S	P/S	P/U	–

*P* Significant positive effect, *N* Significant negative effect, ー: non-significant effect, *U* Under threshold of small effect size, *S* Small effect size, *M* Medium effect size, *L* Large effect size

Excepting fourth graders, the students who reported greater rumination showed greater drive for thinness the following year (*β* = 0.029 − 0.071, *p* < 0.001), with small to medium magnitudes of effects. In addition, students in all grades reporting greater rumination showed more severe binge eating/purging behaviors in the following year (*β* = 0.030–0.035, *p* < 0.001) with small effect sizes.

Regarding the effect of distraction, students in the seventh and eighth grades who reported more frequent use of distraction showed more significant drive for thinness the following year (*β* = 0.016–0.023, *p* < 0.05). Excepting eighth graders, the students with greater distraction showed more severe binge eating/purging behaviors in the following year (*β* = 0.023–0.026, *p* < 0.05). However, these coefficients were below under the criterion for small effect sizes.

In terms of its cross-lagged effects, problem-solving did not significantly explain any levels of either drive for thinness or binge eating/purging behaviors among students in any grade at baseline (drive for thinness: *β* = − 0.009–0.001, *n.s.*, binge eating/purging behaviors: *β* = − 0.009–− 0.007, *n.s.*).

### Cross-lagged effects of associations between AEB and other variables

The cross-lagged panel model indicated that severe drive for thinness significantly predicted greater rumination (*β* = 0.053–0.077, *p* < 0.001) with medium to large effect sizes. However, drive for thinness did not explain the levels of problem-solving and distraction in the following year among students above the fourth grade (problem-solving: = 0.011 − 0.013, *n.s.*, distraction: *β* = 0.015–0.021, *n.s.*). Among all students, except for eighth graders, drive for thinness significantly predicted greater BMI in the following year (*β* = 0.029–0.097, *p*s < 0.001), with small to large effect sizes. Inversely, greater BMI in all grades significantly predicted increased drive for thinness (*β* = 0.086–0.119, *p*s < 0.001), with medium-large effect sizes.

Regarding bulimic symptomatology, fourth to seventh graders with severe binge eating/purging behaviors reported greater rumination the following year (*β* = 0.019–0.028, *p*s < 0.05), but these coefficients were below the threshold for small effect sizes. Fourth to sixth graders with severe binge eating/purging behaviors showed less problem-solving in the following year (*β* = − 0.015–− 0.039, *p*s < 0.05). Further, over-sixth graders with severe binge eating/purging behaviors reported greater distraction (*β* = 0.018–0.027, *p*s < 0.05) in the following year. However, most of these coefficients were below the threshold for small effect sizes. Binge eating/purging behaviors in all grades significantly explained increased BMI in the following year (*β* = 0.022–0.029, *p*s < 0.05), although these coefficients were also below the threshold for small effect sizes. Greater BMI in fourth, fifth, and eighth grades significantly predicted increased binge eating/purging behaviors in the following year (*β* = 0.040–0.060, *p*s < 0.01), with small-medium effect sizes (Table 11).

### Results regarding depressive symptoms

Students in all grades (i.e., fourth to eighth grade) who displayed greater rumination and problem-solving showed more and less severe depressive symptoms in the following years, respectively (rumination: *β* = 0.090–0.127; problem-solving: *β* = − 0.072–− 0.064, *ps* < 0.001), with medium-large effect sizes (Table 10). Students above the fifth grade who displayed greater distraction showed less severe depressive symptoms in the following year (*β* = − 0.027 to − 0.024, *p*s < 0.01), although these coefficients were below the threshold for small effect sizes.

Regarding the effects of AEB, fourth- to seventh-grade students with a stronger drive for thinness experienced more severe depressive symptoms in the following year (*β* = 0.032–0.090, *p*s < 0.001), with effect sizes ranging from small to large. However, binge eating/purging behaviors did not significantly explain the level of depressive symptoms in the following year in any grade (*β* = − 0.018–0.010, *n.s.*).

Regarding the prospective effects of depressive symptoms, all graders with more severe depressive symptoms showed greater rumination in the following year (*β* = 0.083–0.110, *p*s < 0.001), with a large effect size. Moreover, students from all grades with severe depressive symptoms showed less problem-solving and distraction in the following year (problem-solving: *β* = − 0.116–− 0.105, *p*s < 0.001; distraction: *β* = − 0.144–− 0.122, *p*s < 0.001; Table 8 and 9), with large effect sizes. Greater depressive symptoms among students in fourth to seventh grades significantly predicted the severity of drive for thinness and binge eating/purging behaviors in the following year (drive for thinness: *β* = 0.029–0.073, *p*s < 0.01; binge eating/purging behaviors: *β* = 0.021–0.039, *p*s < . 05), with most coefficients indicating small to medium effect sizes.

## Discussion

This study examined the simultaneous effects of three response-style strategies on AEB levels among students in the fourth to eighth grades in Japan. The results of the cross-lagged panel model showed that rumination significantly predicted the severity of AEB in the following year among students in almost all grades, with small to medium effect sizes. However, we found that distraction had a very weak on AEB, while problem-solving had no significant effects on AEB. Furthermore, severe drive for thinness and binge eating/purging behaviors predicted the levels of the three response-style strategies in the following year among students in specific grades.

### Prospective associations between rumination and AEB

Students in all grades who exhibited higher levels of rumination demonstrated an increase in binge eating/purging behaviors in the subsequent year, although the effect sizes were small. This finding is consistent with those of various studies, both meta-analytic and individual [15–17, 48]. Moreover, the current study controlled for potential confounding variables, such as BMI and depressive symptoms, which could influence the relationship between rumination and bulimic symptoms. Based on the collective evidence from this and previous studies, despite the relatively small effect size, it can be concluded that rumination serves as a significant risk factor for an increase in binge eating/purging behaviors during adolescence.

The effect of frequent rumination on increased binge eating/purging behaviors may be explained by the short-term or instantaneous function of rumination. Multiple experimental studies have demonstrated that induced or momentary rumination leads individuals to experience increased negative affect at subsequent occasion [49, 50]. Thus, individuals prone to rumination are more likely to experience a short-term escalation in negative affect daily. The occurrence of binge eating and purging is related to this short-term negative affect. For example, one meta-analysis using ecological momentary assessment found that negative affect precedes binge eating behaviors [51]. Similar findings exist for purging [52, 53]. Considering these empirical findings, the momentary increase in negative affect caused by rumination may help explain its contribution to an increase in binge eating/purging behaviors.

In terms of its impact on anorexic symptoms, greater rumination was found to predict the severity of drive for thinness among students of all grades, except for fourth graders, with effect sizes ranging from small to medium. To the best of our knowledge, this study is the first to demonstrate the positive contribution of rumination to the severity of drive for thinness in a sample of elementary and junior high school students. In a similar vein, recent research by Verschueren et al. [15] reported a positive link between rumination and drive for thinness in a sample of high schoolers. Moreover, previous studies have indicated that individuals with eating pathology or those engaged in extreme dieting tend to exhibit higher levels of rumination than healthy individuals [48]. Notably, this study is also the first to reveal that rumination concurrently predicts the severity of both drive for thinness and binge eating/purging behaviors during early adolescence, thereby enhancing our comprehension of the prospective connections between rumination and abnormal eating behaviors at this life stage.

Furthermore, we observed the significant effects of binge eating/purging behaviors on rumination in the following year among students from almost all grades, indicating a reciprocal relationship between rumination and binge eating/purging behaviors. This result is consistent with those of previous studies [15–17]; however, it is important to note that the effect size was very small. In line with the current study, Verschueren et al. [15] found a small effect of bulimic symptoms on rumination (*β* = 0.04–0.05) using the same cross-lagged panel model as in our study. Since Verschueren et al. [15] did not control for the other two response-style strategies (i.e., problem-solving and distraction), depressive symptoms, or BMI, which are all associated with rumination and bulimic symptoms, it is also understandable that the coefficients of the effect of binge eating/purging behaviors on rumination that we reported was smaller than that found by Verschueren et al. [15]. Consequently, these results suggest that although a reciprocal relationship between rumination and binge eating/purging behaviors may develop in a subset of adolescents, the effects may be small, particularly the effect of binge eating/purging behaviors on rumination.

We observed small to medium effects of drive for thinness on increased rumination across all grades. In contrast, Verschueren et al. [15] reported no significant effects of drive for thinness on rumination. While we cannot fully explain this inconsistency between our study and that of Verschueren et al. [15], one possible reason may be the difference in analytical methodology. We introduced the levels of the three response-style strategies simultaneously into the proposed model, controlling for cohort effects and variables previously reported to be related to AEB, such as BMI and depressive symptoms. In contrast, Verschueren et al. [15] did not control for these variables. The additional procedures in our study may contribute to more robust results regarding the effect of a single response-style strategy. Therefore, the reciprocal relationships of rumination with drive for thinness and binge eating/purging behaviors shown in this study are considered robust and reliable.

Based on the sizes of the effects of drive for thinness and binge eating/purging behaviors on rumination one year later, it appears that a more prominent vicious circle may develop between rumination and drive for thinness than between rumination and binge eating/purging behaviors. The greater effect of drive for thinness compared to binge eating/purging behaviors may be attributed to the function of rumination. Some researchers propose that rumination is a cognitive pursuit aimed at achieving higher-order goals when facing failure in their attainment [54]. In fact, multiple experimental studies have supported this function of rumination [55, 56]. The same cognitive process is considered to develop in adolescents who exhibit body dissatisfaction. Body dissatisfaction results from the deviations between the actual or perceived body shape or weight and the ideal ones that individuals or the culture maintain [57]. Furthermore, body dissatisfaction is related to other anorexic symptoms, such as body checking and restricting dieting [20]. Consistent with this perspective, multiple studies have demonstrated a positive association between rumination and body dissatisfaction [57, 58]. Based on these empirical findings including the present study, the implementation of psychoeducation in school settings regarding developmentally appropriate body shape and weight may prevent adolescents from feeling body dissatisfaction and ruminating about their body shape and weight. Furthermore, given that body dissatisfaction is associated with other anorexia symptoms and bulimic symptoms [20], a reduction in rumination about body shape/weight antipathy, body shape dissatisfaction, may also contribute to the decrease in binge eating/purging behaviors.

### Prospective associations between distraction/problem-solving and AEB

More frequent use of distraction significantly predicted the severity of drive for thinness and binge eating/purging behaviors among students in several grades (Table 5, 6, and 12), although these significant coefficients indicated very small effects (i.e., *β* <|.03|). Partially consistent with this finding, a recent prospective study involving individuals diagnosed with eating disorders, conducted with a one-month interval, revealed that a higher frequency of using distraction during mealtime predicted an increase in binge eating (*β* = 0.13; [24]). Both the current study and that of Vanzhula et al. [24] showed a significant positive effect of distraction on binge eating/purging behaviors; however, given the difference in the effect sizes, it is suggested that limited distraction from foods, rather than generalized distraction, contributes to the increase in binge eating/purging behaviors. Therefore, although several studies have argued that distraction can function as an avoidant strategy that can exacerbate levels of AEB [13, 59, 60], our data suggest that generalized distraction may have little effect on the severity of AEB during adolescence.

There is limited data on the prospective association between problem-solving and AEB during adolescence. In our study, the frequency of problem-solving did not significantly predict the severity of drive for thinness and binge eating/purging behaviors in the following year in any grade. This contrasts with previous findings; for example, one meta-analysis that focused solely on cross-sectional studies [12] and a recent cross-sectional study involving adolescents [13] reported a weak negative association between problem-solving and AEB. It is important to note that causality cannot be established for effects that do not reach statistical significance in a cross-lagged model [61]. Given the lack of significant effects of problem-solving on both drive for thinness and binge eating/purging behaviors in our study, despite its large sample size, it seems plausible that problem-solving makes almost no direct contribution to levels of AEB during adolescence. This suggests that the cross-sectional associations between problem-solving and AEB during adolescence may be superficial.

While this study is the first to investigate the prospective contribution of drive for thinness and binge eating/purging behaviors to the frequency of distraction and problem-solving during adolescence, our findings indicate that AEB showed either no significant effects or only very small effects (i.e., *β* <|.03|) on the frequency of using these strategies in almost all grades. Notably, greater binge eating/purging behaviors only predicted a lower frequency of using the problem-solving strategy in the following year among fourth graders, with a small effect size (Table 8). Explaining why this negative association between binge eating/purging behaviors and problem-solving was observed exclusively in fourth graders is beyond the scope of this study. However, considering the consistently non-significant or small effects of AEB on distraction and problem-solving across most grades, our data suggest that meaningful prospective associations between distraction/problem-solving and AEB may not emerge during adolescence.

### Effects of/on depressive symptoms

This study’s findings also shed light on the effects of and on depressive symptoms. To begin with, severe depressive symptoms predicted higher levels of drive for thinness and binge eating/purging behaviors in the subsequent year among students across nearly all grades. These findings align with clinical studies that have demonstrated the role of addressing depressive symptoms in aiding recovery from eating disorders [62]. As a result, mitigating depressive symptoms becomes crucial in the efforts to prevent the development of AEB among adolescents.

Second, with regard to the impact of AEB on depressive symptoms, it was observed that only drive for thinness predicted the severity of depressive symptoms. This finding is consistent with a prior study involving early adolescents, which found that body dissatisfaction, a key aspect of anorexic symptomatology, can predict the extent of depressive symptoms [63]. In contrast to our findings, various studies have reported a positive prospective association between binge eating/purging behaviors and depressive symptoms [16, 17, 30]. However, it is important to note that these studies did not measure anorexic symptoms. Furthermore, it is worth highlighting that drive for thinness and binge eating/purging behaviors exhibit moderate correlations (Table 3 and 4; [13]). Overall, it is possible that drive for thinness influence the relationship between binge eating/purging behaviors and depressive symptoms, as suggested by previous research. This implies that drive for thinness, but not binge eating/purging behaviors, could prospectively contribute to the emergence or persistence of depressive symptoms.

Third, consistent with prior prospective studies [10, 15], our investigation demonstrated that rumination predicted the severity of depressive symptoms, while problem-solving and distraction strategies contributed to decreased depressive symptoms, except for the effect of distraction in fourth graders. Notably, the effect sizes for rumination were medium to large across various grades. When considered alongside the observed association between higher depressive symptoms and increased AEB in the subsequent year, these findings suggest that the three response-style strategies indirectly contribute to the levels of AEB through their impact on elevating or reducing depressive symptoms.

### Clinical implications

The results of the cross-lagged panel model showed that rumination almost consistently predicted the severity of drive for thinness, binge eating/purging behaviors, and depressive symptoms the following year, even with small effects. In line with previous findings [12], these results suggest that rumination is a significant risk factor contributing to the severity of various psychopathologies. In addition, the auto-regressive coefficients on rumination, which seemed to increase with the upper grades, remained moderate to large (Table 7). These findings imply that a decrease in rumination is critical to alleviating mental health problems during adolescence. Interventions to reduce the level of rumination should be implemented in the early stages of adolescence, specifically for at-risk adolescents for the onset of eating and depressive disorders.

This study provides evidence that distraction can help adolescents alleviate depressive symptoms, which contribute to the severity of AEB. Consistent with this finding, the use of distraction has been shown to assist patients with depressive disorders in managing negative moods [64]. Moreover, distraction has been reported to be more commonly used than other strategies [65]. These empirical results suggest that distraction may be a helpful strategy for interventions or psychological education to alleviate depressive symptoms in adolescents.

However, our findings also provide evidence that distraction exacerbated binge eating/purging behaviors in almost all grades. This is consistent with prior studies, which have also found the use of distraction to exacerbate bulimic symptoms [24]. Therefore, it is necessary to teach adolescents the appropriate way to use distraction: as a temporary measure to regulate adverse effects rather than as a way to avoid stressful situations that individuals can solve themselves.

While CBT-E serves as an effective psychological intervention, fostering improvements in patients' problem-solving skills related to eating behaviors, it may also be associated with a ruminative response-style. CBT-E aims to alter patients' perspectives on body shape, weight, and eating, alongside enhancing their problem-solving abilities [20]. Specifically, patients are educated that their thoughts and images about body shape, weight and eating are symptomatic of their illness. They are encouraged not to unquestionably accept or adhere to these thoughts and images. This education may help interrupt the patients' ruminative processes.

As discussed earlier, considering that rumination involves a goal-oriented cognitive process [54], individuals with eating disorders are likely to ruminate about ways to achieve their higher-order goal, such as their ideal body shape and weight. Nevertheless, with repeated education within the framework of CBT-E that rumination is a part of the symptomatology, patients may begin to prioritize the advice of therapists, gradually placing the content of rumination in the background. Future studies are essential to examine whether CBT-E contributes to attenuating the rumination present in patients with eating disorders. If it is demonstrated that CBT-E indeed leads to a reduction in rumination among these patients, the findings regarding rumination in this study will hold greater clinical significance in explaining the subsequent reduction of AEB.

### Limitations

Despite its contributions, this study has the following limitations. First, it relied on self-reported measurements. Given the evidence indicating that individuals may disclose lower levels of AEB in self-reporting than in interviewed assessments [66], the levels of AEB among the students in the current study may be underestimated. Second, since we based the measurements of the variables, except BMI and SES, on self-reporting, we might observe the confounding effect of shared respondent variance. However, agreement between parent and adolescent reports regarding the measurement of adolescent emotional distress and AEB has been reported to be weak and moderate, respectively [67, 68]. Furthermore, given that response-style strategies involve internal cognitive processes, it may be inappropriate to rely on parental reports of their child’s response-style strategies. Third, the current study did not conduct a diagnostic assessment of eating disorders. Further studies are needed to clarify whether the three response-style strategies that were analyzed simultaneously predict the development of eating disorders during adolescence. Fourth, the variability in BMI values could be a limitation in this study. The sample comprised 4th to 9th graders in early adolescence, where BMI tends to be highly variable [69]. For example, due to the three-month gap between the timing of the physical examination and the annual survey, the students' BMI during the survey period may differ from the BMI measured in the physical examination. Fifth, interpersonal problems may underlie the study’s findings. Previous studies have reported that interpersonal issues are associated with AEB levels [70, 71] and that response-style strategies relate to the severity of an individual’s interpersonal problems [72]. Therefore, further longitudinal studies are needed to examine the prospective associations between AEB, response-style strategies, and interpersonal problems to further clarify the development or maintenance of AEB during adolescence.

### Conclusions

In the current study, we found that greater rumination predicted higher levels of drive for thinness and binge eating/purging behaviors in the following year in almost all grades. Additionally, stronger drive for thinness were linked to an increase in rumination and depression. Furthermore, in line with previous findings, the frequency of using response-style strategies predicted the severity of depressive symptoms. Particularly, we observed a vicious cycle between rumination, drive for thinness, and depressive symptoms. These results expand our understanding of the association between response-style strategies and AEB in adolescents and can contribute to the development of interventions aimed at maintaining mental health in adolescents.

## Data Availability

The datasets generated and analyzed during the current study are available from the corresponding author on reasonable request.

## Abbreviations

AEB: Abnormal eating behaviors/attitudes
CBT-E: Enhanced cognitive behavior therapy
ASCS-Project: Aichi School Cohort Study Project
RSQ-MS: Response Styles Questionnaire for Middle School Students
ABQ-EJ: Abnormal Eating Behavior Questionnaire for Elementary and Junior High School Students
JS-DSRS-C: Japanese short version of the Birleson Depression Self-Rating Scale for Children
BMI: Body mass index
NIMSES: Non-Intrusive Measurement of Socioeconomic Status
SES: Socioeconomic status
